# Bio-Oil from Phototrophic Microorganisms: Innovative Technologies and Strategies

**DOI:** 10.3390/biotech15010011

**Published:** 2026-01-26

**Authors:** Kenzhegul Bolatkhan, Ardak B. Kakimova, Bolatkhan K. Zayadan, Akbota Kabayeva, Sandugash K. Sandybayeva, Aliyam A. Dauletova, Tatsuya Tomo

**Affiliations:** 1 Faculty of Biology and Biotechnology, Al-Farabi Kazakh National University, Al-Farabi 71, Almaty 050038, Kazakhstan; bkenzhegul23@gmail.com (K.B.); sandugash.sandybayeva@kaznu.edu.kz (S.K.S.); aliyusha.dau@mail.ru (A.A.D.); 2 Department of Chemical and Biochemical Engineering, Institute of Geology and Oil-Gas Business Institute Named After K. Turyssov, Satbayev University, Satpaev 22, Almaty 050043, Kazakhstan; 3 Faculty of Natural Sciences, L.N. Gumilyov Eurasian National University, Satpaev 2, Astana 010008, Kazakhstan; akbota.akhmetkali@gmail.com; 4 Institute of Arts and Sciences, Graduate School of Science, Tokyo University of Science, Tokyo 162-8601, Japan; tomo@rs.tus.ac.jp

**Keywords:** phototrophic microorganisms, bio-oil, circular bioeconomy, biorefinery, synthetic biology

## Abstract

The transition to low-carbon energy systems requires scalable and energy-efficient routes for producing liquid biofuels that are compatible with existing fuel infrastructures. This review focuses on bio-oil production from phototrophic microorganisms, highlighting their high biomass productivity, rapid growth, and inherent capacity for carbon dioxide fixation as key advantages over conventional biofuel feedstocks. Recent progress in thermochemical conversion technologies, particularly hydrothermal liquefaction (HTL) and fast pyrolysis, is critically assessed with respect to their suitability for wet and dry algal biomass, respectively. HTL enables direct processing of high-moisture biomass while avoiding energy-intensive drying, whereas fast pyrolysis offers high bio-oil yields from lipid-rich feedstocks. In parallel, catalytic upgrading strategies, including hydrodeoxygenation and related hydroprocessing routes, are discussed as essential steps for improving bio-oil stability, heating value, and fuel compatibility. Beyond conversion technologies, innovative biological and biotechnological strategies, such as strain optimization, stress induction, co-cultivation, and synthetic biology approaches, are examined for their role in tailoring biomass composition and enhancing bio-oil precursors. The integration of microalgal cultivation with wastewater utilization is briefly considered as a supporting strategy to reduce production costs and improve overall sustainability. Overall, this review emphasizes that the effective coupling of advanced thermochemical conversion with targeted biological optimization represents the most promising pathway for scalable bio-oil production from phototrophic microorganisms, positioning algal bio-oil as a viable contributor to future low-carbon energy systems.

## 1. Introduction

The global energy transition toward sustainable and low-carbon systems has intensified the search for renewable and environmentally benign alternatives to fossil fuels. Among the various renewable energy pathways, bio-oil production from phototrophic microorganisms represents a promising and sustainable approach due to its carbon-neutral potential and adaptability to diverse environments. Bio-oil derived from these organisms can substantially reduce greenhouse gas (GHG) emissions, sulfur oxides (SO_x_), nitrogen oxides (NO_x_), and particulate matter while promoting energy security and rural development [1]. In contrast to fossil-based fuels, microbially derived bio-oil integrates carbon capture through photosynthesis, creating a closed carbon loop and contributing directly to climate change mitigation.

Microalgae, a diverse group of photosynthetic microorganisms, have attracted considerable attention as a next-generation biofuel feedstock due to their high lipid content, rapid growth rate, and adaptability to different cultivation environments [2]. Unlike conventional terrestrial crops, microalgae can be cultivated in freshwater, seawater, and wastewater, thereby reducing competition for arable land and freshwater resources [3,4]. Their high photosynthetic efficiency and capacity for carbon dioxide fixation position them as effective biological platforms for sustainable fuel production [5].

Cyanobacteria further expand the potential of phototrophic systems through their metabolic versatility and ability to synthesize a broad spectrum of biofuel precursors. Depending on processing routes, cyanobacterial biomass can be converted into gaseous, liquid, and solid biofuels, supporting the concept of integrated algal biorefineries capable of generating multiple energy carriers from a single biological source [6]. In parallel, a wide range of microalgal taxa has been explored for their lipid accumulation capacity and suitability for bio-oil production, enabling species selection and strain development tailored to specific conversion pathways [7].

Bio-oil from phototrophic microorganisms is predominantly obtained through thermochemical conversion routes, including fast pyrolysis and hydrothermal liquefaction [8]. These approaches enable the transformation of whole algal biomass into liquid fuel intermediates and are considered particularly attractive for large-scale applications [9]. However, algal-derived bio-oils typically contain elevated levels of oxygenated and nitrogen-containing compounds, which negatively affect fuel stability and quality [10]. Consequently, upgrading strategies such as catalytic hydrotreatment and deoxygenation are essential to improve physicochemical properties and achieve fuel characteristics compatible with existing energy infrastructures [11,12].

Despite significant progress in algal cultivation, thermochemical conversion, and upgrading technologies, research on the integrated production of microalgae-derived bio-oil remains fragmented [13,14]. Many studies focus on isolated aspects of the value chain, such as biomass accumulation or conversion efficiency, while issues related to process integration, scalability, and system-level optimization are less comprehensively addressed [15]. In addition, the complex biochemical composition of algal biomass introduces challenges that are not fully captured by conversion models developed for lignocellulosic feedstocks.

In this context, the present review provides a structured and critical assessment of bio-oil production from phototrophic microorganisms, with particular emphasis on innovative thermochemical conversion routes and emerging strategies to enhance fuel quality and process sustainability. By synthesizing current knowledge on biomass characteristics, conversion pathways, upgrading approaches, and system integration, this review aims to identify key technological bottlenecks and research gaps and to support the development of scalable, energy-efficient, and sustainable microalgae-based bio-oil production systems.

## 2. Phototrophic Microorganisms as Advanced Feedstocks for Bio-Oil Production

Phototrophic microorganisms, including microalgae and cyanobacteria, are considered advanced feedstocks for bio-oil production due to their high biomass productivity, favorable biochemical composition, efficient resource utilization, and compatibility with integrated biorefinery systems. In contrast to terrestrial biomass, these organisms provide quantitatively superior areal productivities and biochemical flexibility, which are critical for thermochemical conversion into bio-oil [16].

### 2.1. High Biomass Productivity and Growth Performance

One of the primary advantages of phototrophic microorganisms is their high biomass productivity compared to terrestrial energy crops. Under optimized cultivation conditions, microalgal species such as *Chlorella vulgaris* and *Scenedesmus obliquus* typically achieve areal productivities of 20–30 g m^−2^ day^−1^, while values exceeding 40 g m^−2^ day^−1^ have been reported in high-rate algal ponds and advanced photobioreactor systems [17]. Similarly, *Nannochloropsis* species exhibit high volumetric productivities and are widely used in large-scale outdoor cultivation [18].

Cyanobacteria also demonstrate competitive growth rates. For example, *Spirulina platensis* is known for its rapid biomass accumulation and robustness under alkaline conditions, enabling stable cultivation at an industrial scale [19]. Many phototrophic microorganisms exhibit short doubling times, ranging from several hours to one day, allowing rapid biomass turnover and continuous harvesting strategies. These growth characteristics are particularly advantageous for bio-oil production, as high biomass productivity directly reduces land requirements and improves the feasibility of large-scale thermochemical conversion processes such as hydrothermal liquefaction and pyrolysis [20].

### 2.2. Lipid Accumulation and Biochemical Composition

The suitability of phototrophic microorganisms as bio-oil feedstocks is strongly influenced by their biochemical composition, particularly lipid, protein, and carbohydrate content. Lipid accumulation in microalgae varies widely among species and cultivation conditions, typically ranging from 20 to 50% of dry weight, with values exceeding 60% reported under nutrient stress conditions [21]. Species such as *Chlorella vulgaris*, *Nannochloropsis oculata*, and *Scenedesmus obliquus* are well known for their ability to accumulate triacylglycerols (TAGs), which contribute directly to bio-oil yield during thermochemical conversion [22]. In contrast, *Botryococcus braunii* is distinguished by its capacity to synthesize long-chain hydrocarbons structurally similar to petroleum fractions, making it particularly attractive for direct fuel applications [23].

Cyanobacterial biomass generally contains lower lipid content but higher protein fractions, which influence the nitrogen content of algal bio-oil [24]. While this may necessitate additional upgrading steps, protein-rich biomass can enhance overall carbon recovery during thermochemical processing. The combined presence of lipids, carbohydrates, and proteins allows whole-cell conversion approaches, eliminating the need for prior biochemical fractionation [25].

### 2.3. Resource Efficiency and Wastewater-Based Cultivation

Phototrophic microorganisms offer significant advantages in terms of resource efficiency, as they can be cultivated using non-arable land and alternative water sources. Numerous studies have demonstrated successful microalgal growth in municipal, agricultural, and industrial wastewater, where nitrogen and phosphorus concentrations support robust biomass production [26].

Species such as *Chlorella vulgaris*, *Scenedesmus obliquus*, and *Auxenochlorella protothecoides* have shown nutrient removal efficiencies of up to 80–90% for nitrogen and 70–85% for phosphorus, while simultaneously producing lipid-rich biomass suitable for bio-oil conversion [27]. Wastewater-grown microalgae typically exhibit slightly lower lipid content compared to nutrient-replete cultures; however, the substantial reduction in cultivation costs and freshwater demand offsets this limitation [28].

The integration of wastewater treatment with microalgal cultivation thus provides a dual benefit: environmental remediation and low-cost feedstock generation, which is particularly relevant for the economic viability of algal bio-oil systems [29].

### 2.4. Carbon Fixation and CO_2_ Utilization

Phototrophic microorganisms exhibit high carbon dioxide fixation capacities, converting inorganic carbon into organic biomass through photosynthesis. It has been reported that microalgae can assimilate approximately 1.6–1.8 kg of CO_2_ per kg of dry biomass produced, significantly exceeding the carbon fixation efficiency of terrestrial plants on an areal basis [30].

Certain cyanobacteria possess carbon-concentrating mechanisms (CCMs) that enable efficient CO_2_ uptake even at low ambient concentrations. This feature allows the direct utilization of flue gas or industrial CO_2_ streams as carbon sources for biomass production. The resulting carbon-rich biomass serves as an effective precursor for bio-oil generation, linking carbon capture with renewable fuel production [31].

### 2.5. Compatibility with Integrated Biorefinery Systems

Beyond bio-oil production alone, phototrophic microorganisms are well suited for integrated biorefinery concepts. Their biomass can be converted into multiple energy carriers and value-added products, including bio-oil, biodiesel, bioethanol, biohydrogen, and biogas. Residual streams generated during thermochemical conversion, such as aqueous phases and solid residues, can be recycled for nutrient recovery or energy generation [32].

This multiproduct capability enhances overall process efficiency and economic resilience. For example, coupling bio-oil production with the recovery of proteins or carbohydrates for feed or biochemical applications can significantly improve the techno-economic performance of algal biorefineries [33].

**Figure 1:**
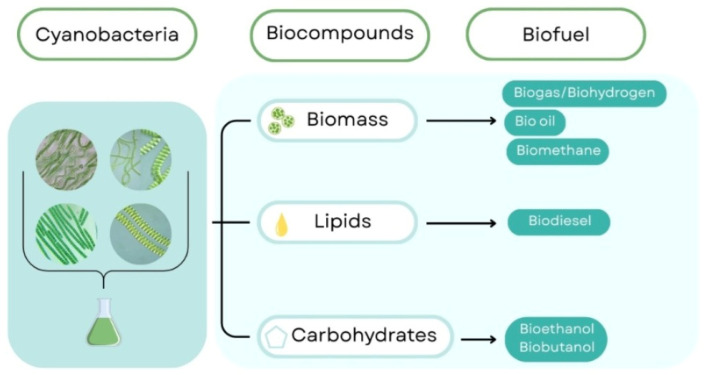
Schematic overview of major metabolic products and biofuel pathways derived from cyanobacteria. Cyanobacterial biomass is fractionated into main biochemical components, including total biomass, lipids, and carbohydrates. Arrows indicate conversion pathways to different biofuel products: biomass to biogas/biohydrogen, bio-oil, and biomethane; lipids to biodiesel; carbohydrates to bioethanol and biobutanol. This figure is a creation by the authors, developed for this manuscript based on literature data [34,35,36]. The figure was created using Canva (https://www.canva.com/).

Phototrophic microorganisms, particularly cyanobacteria, exhibit remarkable metabolic versatility that enables the synthesis of a wide range of energy carriers and value-added compounds. Through photosynthetic carbon fixation, these organisms convert inorganic carbon into biomass components that can subsequently serve as precursors for multiple biofuel pathways. Depending on the targeted fraction of the biomass, distinct biochemical and thermochemical conversion routes can be applied to generate gaseous, liquid, and solid biofuels. As illustrated in Figure 1 for cyanobacteria, phototrophic microorganisms can be fractionated into major biochemical pools, including lipids, carbohydrates, and proteins, enabling the production of bio-oil, biodiesel, bioethanol, and biohydrogen (Figure 1).

As illustrated in Figure 1, cyanobacterial biomass can be fractionated into major biochemical pools, including whole biomass, lipids, and carbohydrates, each of which can be directed toward specific biofuel production routes. Whole biomass can be converted into biogas, biohydrogen, bio-oil, or biomethane, whereas lipid fractions are primarily utilized for biodiesel production. In parallel, carbohydrate-rich fractions serve as substrates for bioethanol and biobutanol generation. This multiplicity of conversion pathways highlights the suitability of phototrophic microorganisms for integrated, multi-output bioenergy systems [37].

The metabolic diversity and flexible conversion potential of phototrophic microorganisms provide a strong foundation for the development of integrated biofuel production systems. Their ability to generate multiple bioenergy carriers from a single biological platform distinguishes them from conventional feedstocks and supports their role in advanced biorefinery concepts [38]. These characteristics position microalgae and cyanobacteria as strategic feedstocks for bio-oil-centered biorefineries, forming a logical basis for the subsequent discussion of cultivation strategies, conversion pathways, and system integration.

## 3. Conversion Pathways for Bio-Oil Production from Phototrophic Microorganisms

The conversion of phototrophic microbial biomass, primarily microalgae and cyanobacteria, into liquid bio-oil represents one of the most promising routes for producing renewable, energy-dense fuels [39]. Due to their high lipid, protein, and carbohydrate content, these organisms can be efficiently transformed into bio-oil and other valuable hydrocarbons through various thermochemical and biochemical methods [40]. Among these, hydrothermal liquefaction (HTL), pyrolysis, and catalytic hydroprocessing (HDO) have shown significant potential for sustainable and large-scale implementation. Additionally, advances in biotechnology and genetic engineering have enabled targeted improvements in biomass composition and conversion efficiency [41].

In the context of algal biofuel production, cultivation strategies and biomass engineering approaches represent upstream optimization steps aimed at improving biomass yield and composition. In contrast, thermochemical processes such as hydrothermal liquefaction (HTL) and pyrolysis constitute downstream conversion technologies that transform the produced biomass into bio-oil and other fuel intermediates.

### 3.1. Hydrothermal Liquefaction (HTL)

**Figure 2:**
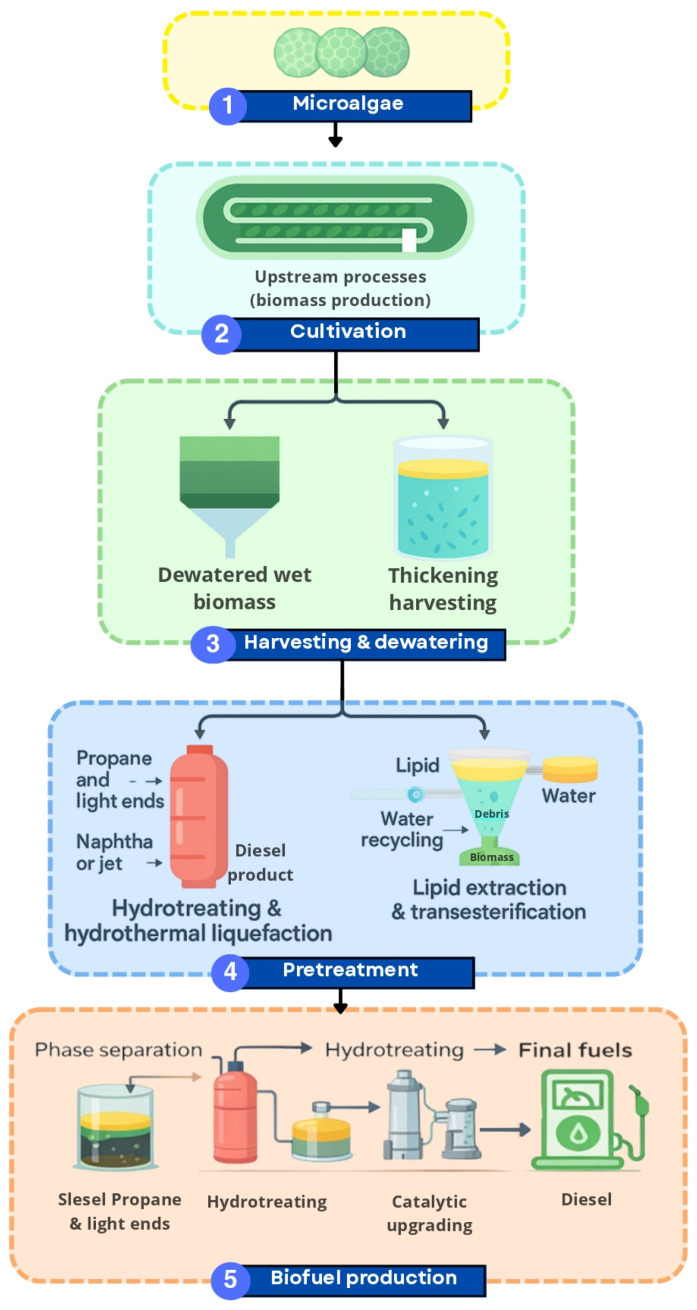
Schematic overview of the microalgae-based biofuel production chain, highlighting hydrothermal liquefaction (HTL) as a distinct thermochemical conversion step. Upstream processes include microalgal cultivation and biomass harvesting, whereas HTL is specifically applied to wet biomass and followed by phase separation. Downstream operations such as hydrotreating and catalytic upgrading convert HTL-derived biocrude into final liquid fuels. This figure is a creation by the authors, developed for this manuscript based on literature data [43]. The figure was created using Canva (https://www.canva.com/).

Hydrothermal liquefaction (HTL) is one of the most efficient thermochemical methods for converting wet algal biomass into bio-oil under subcritical or supercritical water conditions. Unlike pyrolysis, which requires dry biomass, HTL directly processes wet biomass, eliminating the energy-intensive drying step [42]. The overall workflow of the hydrothermal liquefaction process is illustrated in Figure 2, which summarizes the integrated steps from microalgal cultivation to biofuel production. This schematic highlights the closed-loop nature of HTL systems, emphasizing the potential of wet biomass conversion, water recycling, and sustainable biofuel generation.

The key advantage of HTL lies in its compatibility with high-moisture biomass such as microalgae and cyanobacteria, which contain 70–90% water. This enables high yields of bio-crude with energy contents up to 46 MJ/kg and maximum conversion efficiencies around 60% under optimized conditions [44]. The resulting bio-oil typically contains nitrogen- and oxygen-rich compounds derived from proteins and lipids, which can later be upgraded to high-quality transportation fuels.

Pretreatment of biomass prior to HTL, whether mechanical, thermal, or chemical, can improve the accessibility of cell components and enhance conversion efficiency. For lignocellulosic or mixed biomass, pretreatment also increases hydrolysis and fermentation efficiency [45]. HTL enables rapid thermochemical conversion at elevated temperatures, mimicking natural geological processes of crude oil formation but on a timescale of hours rather than millennia.

Optimization of operating conditions, such as temperature, solid loading, and residence time, along with the use of heterogeneous catalysts, has improved both yield and quality of the produced oil [46]. Novel catalysts, such as transition metal oxides and zeolites, help reduce oxygen and nitrogen content, thereby increasing the heating value and stability of the bio-oil. Response Surface Methodology (RSM) approaches have further refined these operational parameters, leading to reproducible and efficient outcomes. Additionally, studies have investigated the life cycle and techno-economic performance of algal HTL systems, demonstrating that recycling aqueous and gaseous by-products can enhance both process sustainability and overall yield [47].

Hydrothermal liquefaction has therefore emerged as a key technology for converting wet algal biomass into high-grade bio-crude. Although energy-intensive harvesting and dewatering remain challenges [47], ongoing research into integrated systems, including electromagnetic and ultrasonic separation, as well as co-production of high-value co-products, is expected to improve the economic feasibility of algal-based biorefineries.

### 3.2. Pyrolysis

Pyrolysis represents another major thermochemical pathway for converting microalgal biomass into bio-oil, gases, and biochar. It involves the thermal decomposition of organic material in the absence of oxygen at elevated temperatures [48]. Depending on heating rate and residence time, pyrolysis can be classified into slow, fast, or flash types. Slow pyrolysis, with prolonged residence times and low heating rates, maximizes char formation, while fast pyrolysis, operating at 450–600 °C with heating rates of 100–1000 °C/s, favors bio-oil production [49].

Optimized fast pyrolysis of lipid-rich algal biomass can yield bio-oil in the range of 50–60% (dry basis). For instance, Li et al. [9] achieved a 62% yield from *Chlorella* sp. at 550 °C, while Brennan et al. [50] obtained 53% using fluidized-bed pyrolysis of lipid-extracted *Chlorella vulgaris*. However, at temperatures exceeding 700 °C, gas production increases at the expense of oil yield.

Various reactor designs have been developed for pyrolysis, including tubular, fluidized-bed, microwave-assisted, and ablative reactors. Fluidized-bed configurations are particularly favored for their excellent heat transfer and scalability. Vacuum systems, on the other hand, allow for rapid vapor condensation and improved oil recovery. Microwave-assisted pyrolysis, though less mature, has shown potential; one study reported oil yields of approximately 28% at 750 W [51].

The use of catalysts such as zeolites (HZSM-5), zirconia, and alumina promotes cracking and deoxygenation of volatile intermediates, generating aromatic hydrocarbons such as benzene, toluene, and xylene (BTX compounds). Bruce et al. [52] demonstrated that catalytic pyrolysis using HZSM-5 at 550 °C transformed a significant portion of algal bio-oil into light aromatics (~36% of organic products). However, catalytic systems often reduce overall oil yield while improving quality.

Process innovations such as sequential lipid extraction followed by pyrolysis of the residual biomass have yielded cleaner bio-oils with lower nitrogen and PAH content. For instance, experiments using *Desmodesmus* sp. cultivated in wastewater produced bio-oils enriched in aliphatic hydrocarbons. Current research focuses on optimizing catalyst composition, temperature, and reactor design to improve yield, stability, and economic viability of pyrolysis-derived algal oils [53].

### 3.3. Catalytic Hydroprocessing (HDO)

Because pyrolysis and HTL oils are rich in oxygenated and nitrogenous compounds, they require upgrading to improve fuel stability and calorific value. Catalytic hydroprocessing, or hydrodeoxygenation (HDO), is a crucial step in refining raw bio-oils into high-quality hydrocarbon fuels. This process involves reacting bio-oil with hydrogen gas at 200–400 °C and pressures exceeding 30 bar in the presence of catalysts to remove oxygen in the form of H_2_O, CO, or CO_2_ [54].

Common catalysts include sulfides Ni–Mo and Co–Mo supported on alumina, as well as noble metals (Pt, Pd, Ru) and advanced materials such as metal carbides, phosphides, and zeolites. These facilitate both hydrogenation and C–O bond cleavage, essential for deoxygenation [55]. For example, Derawi et al. [56] achieved over 80% oxygen removal from *Chlorella*-derived pyrolysis oil using a Ni–Cu/ZrO_2_ catalyst (15% Ni, 6% Cu) at 350 °C and 20 bar H_2_. Multi-stage hydroprocessing, involving mild and severe deoxygenation steps, prevents excessive cracking and enhances product yield. Emerging approaches include hydrogen-donor solvents and in situ hydrogen generation via aqueous-phase reforming, reducing dependence on external hydrogen sources.

HDO processes not only improve fuel properties but also reduce heteroatom content, resulting in hydrocarbons with high energy density, lower viscosity, and improved stability. These upgraded oils are suitable for use in transportation, aviation, and as feedstocks for the petrochemical industry. A comparative overview of bio-oil production methods from phototrophic microorganisms is summarized in Table 1.

As shown in Table 1, hydrothermal liquefaction (HTL) remains the most suitable route for wet algal biomass, whereas pyrolysis and catalytic hydroprocessing offer complementary benefits in terms of product yield and fuel quality. Biotechnological improvements further enhance the feedstock’s energy potential, supporting an integrated approach to microalgae-based biorefineries [66].

### 3.4. Biotechnological Approaches and Genetic Engineering

In addition to thermochemical conversion routes, biotechnological and process intensification strategies play a critical role in enhancing the yield, quality, and overall feasibility of bio-oil production from phototrophic microorganisms. These approaches act primarily at the upstream (biomass composition), midstream (pretreatment and extraction), and downstream (fuel stabilization) levels, thereby complementing thermochemical pathways such as HTL and pyrolysis.

#### 3.4.1. Genetic and Metabolic Engineering of Phototrophic Microorganisms

Genetic and metabolic engineering strategies have been widely explored to enhance lipid accumulation and tailor biomass composition toward bio-oil precursors. Targeted modifications of lipid biosynthesis pathways, stress response mechanisms, and carbon flux distribution have demonstrated substantial improvements in triacylglycerol (TAG) productivity. For example, overexpression of S-adenosylmethionine synthetase (SAMS) in *Chlamydomonas reinhardtii* resulted in a 1.5–2-fold increase in growth and lipid accumulation under nitrogen-limited conditions, while introduction of the transcription factor HSbZIP1 into *Chlorella* sp. HS2 nearly doubled fatty acid content [67].

Further metabolic interventions, including overexpression of acyl-activating enzymes and suppression of long-chain acyl-CoA synthase, yielded up to 142% increases in lipid accumulation, accompanied by a 45% reduction in starch synthesis. Similarly, enhanced expression of ACCase and DGAT genes, combined with inhibition of starch biosynthesis and heterologous gene incorporation, significantly improved total lipid productivity [68]. The application of CRISPR–Cas technologies has enabled precise genome editing in several microalgal species, accelerating strain optimization and expanding the potential for rational biofuel-oriented design.

#### 3.4.2. Co-Cultivation and Biological Conversion Pathways

Co-cultivation strategies have emerged as a promising alternative to monoculture systems by improving nutrient utilization, biomass productivity, and lipid accumulation. A notable example is the co-cultivation of *Chlamydomonas reinhardtii* with the nitrogen-fixing bacterium *Azotobacter chroococcum*, which resulted in a 19-fold increase in lipid productivity (~142 mg L^−1^ day^−1^) compared to monocultures [69]. Such synergistic interactions enhance carbon and nitrogen availability while stabilizing cultivation systems.

Beyond lipid-based pathways, carbohydrate-rich algal biomass can be converted into bioethanol and biohydrogen through biological routes. Enzymatic or acid hydrolysis of starch-rich Chlorella vulgaris biomass yielded 0.46 g glucose g^−1^ biomass, enabling ethanol production of 11.7 g L^−1^, corresponding to approximately 88% of the theoretical yield during fermentation [70]. Dark fermentation of algal hydrolysates has further demonstrated hydrogen yields of up to 958 mL H_2_ g^−1^ volatile solids, highlighting the flexibility of biological conversion pathways [71].

#### 3.4.3. Pretreatment and Extraction Intensification

Despite advances in strain engineering, downstream processing of microalgal biomass remains a major bottleneck due to energy-intensive drying and lipid extraction requirements. Conventional extraction methods, including mechanical disruption, solvent extraction, supercritical CO_2_, and enzyme-assisted techniques, are often associated with high energy demand and operational costs [69].

To address these limitations, pretreatment technologies such as hydrodynamic and acoustic cavitation have gained increasing attention. Cavitation induces cell wall disruption through localized high shear forces and transient high temperatures, significantly enhancing the accessibility of intracellular lipids, carbohydrates, and proteins [55]. Studies have shown that cavitation-assisted pretreatment improves lipid extraction efficiency while reducing solvent usage and processing time, aligning with green chemistry principles [72]. Cavitation intensity can be tuned from mild (~5 MPa) to high (~100 MPa), enabling selective cell wall disruption while preserving sensitive bioactive compounds such as proteins and carotenoids [73]. Although promising, challenges related to scale-up and energy efficiency remain.

#### 3.4.4. Wet Biomass Processing and Fuel Stabilization Strategies

Direct processing of wet biomass, particularly via hydrothermal liquefaction, has been emphasized as a key strategy for improving overall energy efficiency by eliminating the drying step [74]. Recent developments such as microwave-assisted hydrothermal liquefaction (MA-HTL) enable rapid and volumetric heating of wet biomass, resulting in improved bio-oil yield and calorific value compared with conventional HTL [75,76]. MA-HTL has also been applied to non-algal feedstocks such as brewer’s spent grains, demonstrating its versatility for producing value-added chemicals and biofuels.

Table 2 outlines how both biological and process-level interventions can substantially enhance bio-oil production from phototrophic microorganisms. Genetic and metabolic engineering primarily improve lipid accumulation and biomass composition, thereby increasing the availability of bio-oil precursors. In contrast, process-intensification strategies such as cavitation pretreatment, microwave-assisted hydrothermal liquefaction, and supercritical alcohol blending mainly target downstream efficiency by facilitating biomass disruption, improving conversion yields, and enhancing fuel stability. The integration of these complementary strategies offers a promising pathway toward scalable and economically viable algal bio-oil production within advanced biorefinery frameworks.

Other strategies involve utilizing formic acid or dimethyl ether (DME) as solvents to enhance lipid breakdown and improve oil yield [85]. Simulation studies have shown that intensified systems that recycle heat and solvents during the extraction process can cut energy use by over 50% compared to traditional methods. In addition to conversion efficiency, stabilization of algal bio-oil remains a critical challenge due to its high oxygen content and chemical instability. Blending bio-oil with short-chain alcohols, including methanol, ethanol, and isopropanol, under supercritical conditions has emerged as an effective strategy to reduce acidity, increase ester content, and improve higher heating value (HHV) [86]. Among these solvents, isopropanol blending produced the most stable and energy-dense biofuel with balanced physicochemical properties [87]. These blending strategies can be further integrated with catalytic upgrading processes, such as hydrodeoxygenation, to enhance long-term storage stability and combustion performance [88].

**Figure 3:**
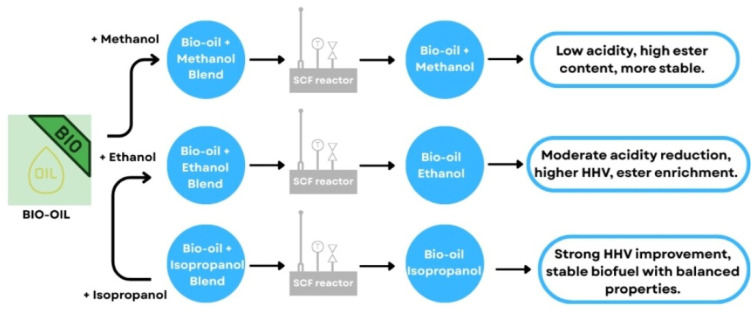
Effect of supercritical alcohol blending on the physicochemical stability of bio-oil. Bio-oil is blended with methanol, ethanol, or isopropanol and processed in a supercritical fluid (SCF) reactor. Arrows indicate the conversion pathway, while the resulting annotations summarize changes in acidity, ester content, higher heating value (HHV), and overall stability of the upgraded bio-oil. This figure is an original creation by the authors, developed for this manuscript based on literature data [89,90]. This figure is an original creation by the authors, developed for this manuscript based on literature data [91,92]. The figure was created using Canva (https://www.canva.com/).

Nevertheless, the high moisture content of microalgal biomass and the instability of the resulting bio-oil (due to its high oxygen content) remain major obstacles. Blending bio-oil with short-chain alcohols, such as methanol, ethanol, or isopropanol, has emerged as an effective approach to improve fuel stability, heating value, and storage performance through esterification and reduction in acidity [88], as schematically illustrated in Figure 3.

Blending algal bio-oil with short-chain alcohols under supercritical conditions effectively reduces acidity, increases ester content, and improves higher heating value (HHV). Among the evaluated solvents, isopropanol provides the most pronounced enhancement in physicochemical stability and energy density, while methanol and ethanol offer moderate improvements in acidity and fuel balance. These stabilization strategies can be readily combined with catalytic upgrading processes, such as hydrodeoxygenation, to further improve long-term storage stability and combustion performance [34].

Overall, bio-oil production from phototrophic microorganisms relies on the synergistic integration of thermochemical conversion routes and targeted biotechnological and process-intensification strategies. Hydrothermal liquefaction remains the most suitable pathway for wet algal biomass, while pyrolysis-based routes provide high liquid yields from dry or lipid-enriched feedstocks. Catalytic hydroprocessing and fuel stabilization techniques are essential to achieve fuel-quality compliance, whereas genetic engineering, co-cultivation, and pretreatment intensification enhance biomass composition and downstream efficiency [93]. Together, these complementary approaches form the technological foundation for scalable and sustainable algal bio-oil production within advanced biorefinery frameworks.

## 4. Integration of Wastewater Streams into Algal Biofuel Production Systems

The integration of wastewater treatment with algal biofuel production represents a critical system-level strategy for improving the sustainability and economic feasibility of algal biorefineries. Unlike conventional cultivation systems that rely on synthetic fertilizers and freshwater, microalgae can efficiently utilize municipal, agricultural, and industrial wastewaters as sources of nitrogen, phosphorus, carbon, and trace elements, thereby coupling biomass production with environmental remediation [89,90,94].

**Figure 4:**
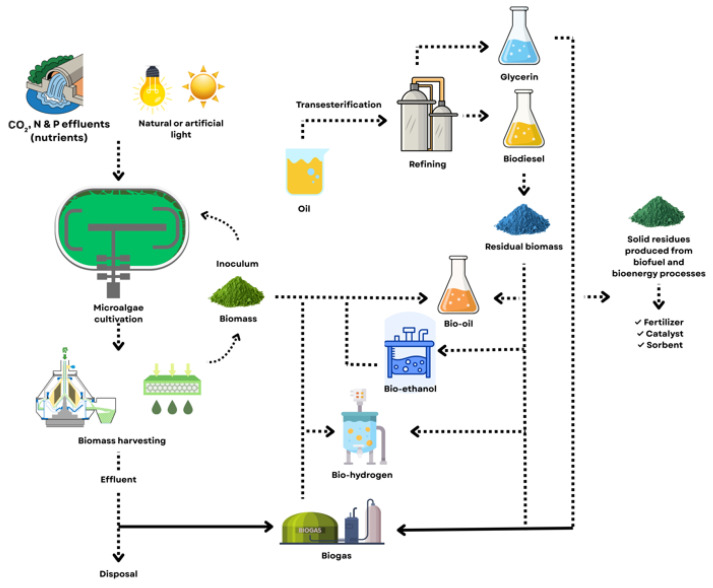
Integration of wastewater streams into microalgae-based biorefinery systems. Wastewater-derived nutrients (CO_2_, N, and P) and natural or artificial light support microalgal cultivation, followed by biomass harvesting. The harvested biomass can be processed through multiple conversion pathways, including biochemical routes (e.g., bioethanol and biohydrogen production), chemical routes (e.g., lipid extraction and transesterification to biodiesel), and thermochemical routes (e.g., hydrothermal liquefaction and pyrolysis to bio-oil). Residual biomass and digestate streams can be further valorized into biogas, fertilizers, catalysts, or sorbents, enabling closed-loop material and energy recycling within a circular biorefinery framework. This figure is a creation by the authors, developed for this manuscript based on literature data [43,97]. The figure was created using Canva (https://www.canva.com/).

Within wastewater-based systems, microalgal biomass can be converted into bioenergy through biochemical, chemical, and thermochemical pathways. Among these, thermochemical routes, particularly hydrothermal liquefaction (HTL) and pyrolysis, are especially attractive, as they enable direct conversion of whole biomass into bio-oil without prior drying or lipid extraction, significantly reducing process energy demand [95,96]. The integration of wastewater utilization, algal cultivation, and downstream biofuel conversion pathways is schematically illustrated in Figure 4.

As depicted in Figure 4, wastewater streams serve as a dual resource by supplying essential nutrients for microalgal growth while simultaneously enabling wastewater treatment. Following cultivation and harvesting, the resulting algal biomass can be directed toward different conversion pathways depending on its biochemical composition and processing objectives. Lipid-rich fractions are primarily converted into biodiesel via transesterification, whereas whole biomass can be utilized for thermochemical conversion to bio-oil or biochemical conversion to bioethanol and biohydrogen. Residual biomass and process by-products, such as digestate and solid residues, are further recycled into biogas, fertilizers, or functional materials, thereby minimizing waste generation and enhancing overall system efficiency.

From an environmental perspective, wastewater-based algal cultivation offers a dual benefit. First, microalgae assimilate dissolved nutrients, primarily nitrogen and phosphorus, thereby mitigating eutrophication and improving effluent quality. Second, algal systems capture CO_2_ from flue gases or dissolved inorganic carbon in wastewater, contributing to carbon mitigation and biological carbon capture [98]. As a result, wastewater-integrated algal biorefineries operate as closed-loop systems consistent with circular economy principles, where waste streams are transformed into valuable resources.

Operational studies demonstrate that nutrient removal efficiencies can reach up to 90% for nitrogen and 80% for phosphorus, depending on wastewater composition, cultivation conditions, and species selection [99]. Microalgal strains such as *Chlorella vulgaris*, *Scenedesmus obliquus*, and *Auxenochlorella protothecoides* have shown particular robustness under variable wastewater conditions, combining effective nutrient removal with high biomass productivity (Table 3).

Despite these advantages, large-scale implementation remains challenged by fluctuations in wastewater composition, the presence of inhibitory compounds, and the energy intensity of biomass harvesting and downstream processing [103]. Current research therefore focuses on advanced photobioreactor designs, hybrid cultivation systems combining open ponds and closed reactors, automated nutrient monitoring, and co-cultivation strategies with bacteria to stabilize microbial consortia and enhance nutrient uptake [42].

Wastewater-integrated algal biofuel systems represent one of the most promising pathways toward carbon-neutral and resource-efficient bioenergy production. By simultaneously addressing wastewater treatment, nutrient recovery, and renewable fuel generation, these systems embody the “waste-to-value” concept central to sustainable biorefinery development.

## 5. Synthetic Biology Strategies for Enhanced Biofuel Yields

Building upon the biotechnological approaches discussed in the previous section, synthetic biology represents a transformative framework for improving biofuel yields from phototrophic microorganisms such as microalgae and cyanobacteria. Unlike conventional genetic engineering, which focuses on the manipulation of single genes, synthetic biology employs a systems-level approach to design and construct new metabolic pathways, regulatory circuits, and cellular modules that enhance the productivity, stability, and resilience of these photosynthetic systems [104].

By integrating metabolic modeling, enzyme optimization, and computational design, synthetic biology allows precise control over photosynthetic efficiency, carbon fixation, and energy partitioning, thereby improving the conversion of CO_2_ into energy-dense lipids and hydrocarbons [105]. Recent research demonstrates that coupling artificial metabolic control with high-throughput omics and CRISPR-based regulation can dynamically redirect carbon fluxes toward lipid biosynthesis and biomass growth under varying environmental conditions [106].

**Figure 5:**
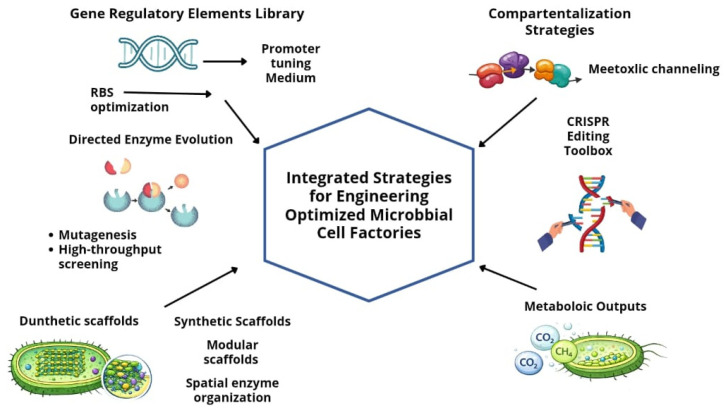
Key molecular and cellular strategies employed in synthetic biology for metabolic reprogramming of phototrophic microorganisms. The schematic highlights integrated approaches for engineering optimized microbial cell factories, including gene regulatory element libraries (e.g., promoter tuning and ribosome binding site optimization), directed enzyme evolution (mutagenesis and high-throughput screening), synthetic and modular scaffolds for spatial enzyme organization, compartmentalization strategies enabling metabolic channeling, CRISPR-based genome editing toolboxes, and the resulting enhancement of metabolic outputs such as CO_2_ fixation and biofuel precursor synthesis. This figure is an original creation by the authors, developed for this manuscript based on literature data [107,108]. The figure was created using Canva (https://www.canva.com/).

Figure 5 illustrates the integration of molecular and cellular strategies used to optimize phototrophic microorganisms as microbial cell factories. Gene regulatory element libraries enable fine control of transcriptional and translational processes, while directed enzyme evolution and synthetic scaffolds enhance catalytic efficiency and metabolic flux distribution. Compartmentalization and metabolic channeling strategies reduce intermediate losses and undesired side reactions, whereas CRISPR-based genome editing provides precise and scalable tools for pathway rewiring. Collectively, these approaches enable coordinated optimization of carbon fixation, energy allocation, and biofuel precursor synthesis in phototrophic systems.

### 5.1. Systems-Based Metabolic Design

Phototrophic microorganisms possess highly flexible metabolisms, making them ideal platforms for synthetic biology-driven redesign. A systems-based design approach enables scientists to reconfigure metabolic fluxes, improve carbon partitioning, and balance energy flow between photosynthesis and biomass accumulation [109].

The development of promoter libraries and enzyme redesign enables fine-tuning of key nodes such as acetyl-CoA carboxylation, malic enzyme activity, and fatty acid elongation, leading to enhanced lipid synthesis. Compartmentalization strategies, localizing biosynthetic enzymes within thylakoid membranes or carboxysomes, reduce energy dissipation and photorespiration losses, thus improving the overall efficiency of carbon assimilation [110].

Synthetic scaffolds that spatially organize catalytic enzymes further accelerate metabolic reactions and improve substrate channeling. For example, *Chlorella vulgaris* and *Synechocystis* sp. engineered to overexpress acetyl-CoA carboxylase (ACCase) and diacylglycerol acyltransferase (DGAT) achieved up to 2.5-fold higher lipid accumulation under nitrogen-limited conditions [111]. Such strategies collectively enable the conversion of solar energy into lipid-based biofuel precursors in programmable phototrophic cells.

### 5.2. Process Optimization and Bio-Oil Upgrading

Beyond cellular-level optimization, synthetic biology increasingly intersects with process engineering to enhance downstream conversion efficiency. Advances such as microwave-assisted hydrothermal liquefaction enable rapid and uniform heating of wet biomass, improving bio-oil yield and energy recovery while eliminating the need for drying [112].

In addition, solvent-based stabilization strategies, including supercritical alcohol blending, have been shown to significantly improve the physicochemical stability and heating value of algal bio-oil, facilitating its integration with catalytic upgrading processes [113]. These combined biological and process-level interventions highlight the importance of co-optimizing biomass composition and conversion technologies.

### 5.3. Integration of Synthetic Biology and Process Engineering

**Figure 6:**
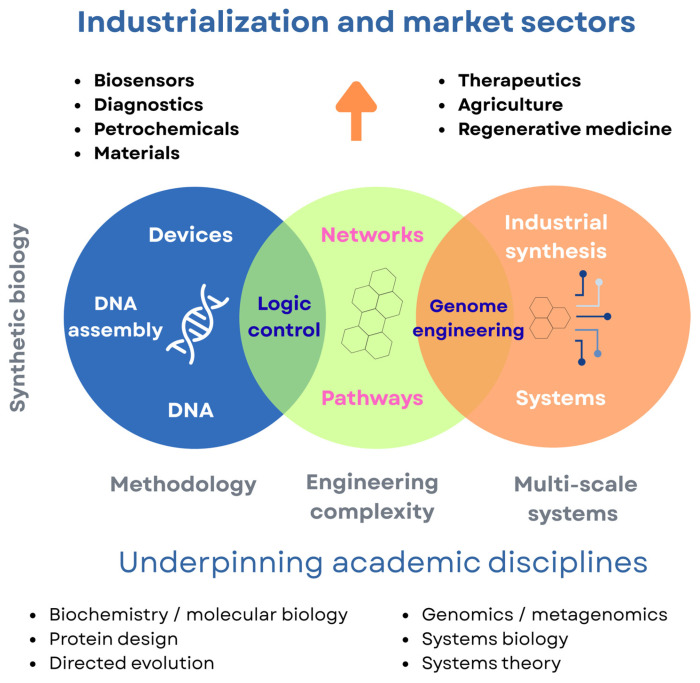
Conceptual framework of synthetic biology as a convergent discipline linking biological design and process engineering. This figure is an original creation by the authors, developed specifically for this manuscript based on published literature data [115,116,117,118]. The arrows indicate the flow of information and integration between biological design, engineering complexity, and industrial applications, while the colors represent different functional domains within synthetic biology. This figure is an original creation by the authors, developed specifically for this manuscript based on published literature data [119,120,121,122]. The figure was created using Canva (https://www.canva.com/).

Synthetic biology acts as both a platform technology for molecular reprogramming and a translational tool connecting biological design with industrial process engineering. It combines computational modeling, AI-guided pathway optimization, and process intensification to improve both upstream (cellular metabolism) and downstream (conversion and upgrading) stages of biofuel production [114], as illustrated in Figure 6.

Advances in adaptive photobioreactor systems, equipped with real-time sensors and automated feedback loops, enable dynamic control of nutrient inputs, light intensity, and CO_2_ concentration, leading to a 25–40% increase in biomass productivity and lipid yield compared with static cultivation [123]. Machine learning algorithms such as gradient boosting and neural networks have been successfully employed to predict bio-oil yields with R^2^ ≈ 0.9, identifying feedstock composition and reaction temperature as critical parameters [124].

Globally, synthetic biology is emerging as a cornerstone of the circular bioeconomy. Its application in renewable energy, wastewater valorization, and green chemistry is expanding at an annual rate of approximately 19%. The global synthetic biology market is projected to reach USD 54.3 billion by 2029, up from USD 21.1 billion in 2024, reflecting the growing industrial adoption of bio-based systems for sustainable fuel and carbon-neutral technologies [125].

### 5.4. Future Perspectives

Synthetic biology offers an integrative and flexible framework for optimizing the performance of phototrophic microorganisms in bio-oil production. By merging principles of systems design, process engineering, and sustainability science, researchers can address the key limitations of current biofuel technologies, namely, low conversion efficiency, instability of bio-oil, and high production costs [126].

Future progress in synthetic biology-driven algal biofuels will depend on integrating metabolic engineering with data-driven control systems, real-time monitoring, and machine learning-based optimization. Hybrid systems employing synthetic microbial consortia may further enhance robustness, nutrient utilization, and process stability under industrial conditions [115].

## 6. Algal Biofuels Within the Circular Bioeconomy Framework

Global energy shortages, food insecurity, and environmental degradation are among the most critical challenges of the 21st century, largely resulting from unsustainable production systems and extensive dependence on fossil fuels [116]. The combustion of fossil-derived fuels continues to contribute significantly to greenhouse gas (GHG) emissions, leading to climate change, air pollution, and depletion of natural resources. Therefore, the development of bio-based circular systems has become a central priority in achieving the Sustainable Development Goals (SDGs) 2030, particularly those related to clean energy, responsible consumption, and climate action [117].

Microalgae and cyanobacteria offer a sustainable solution within this context due to their remarkable capacity to convert CO_2_, sunlight, and nutrients, including those recovered from wastewater, into valuable biomass components such as lipids, carbohydrates, and proteins [118]. Their productivity per unit area surpasses that of terrestrial crops by several orders of magnitude, making algal systems particularly suitable for the circular bioeconomy [127]. Beyond biofuel production, algal biomass can be valorized into bioplastics, nutraceuticals, feed additives, and biostimulants, supporting integrated biorefinery concepts that maximize value generation from a single biological feedstock [128].

**Figure 7:**
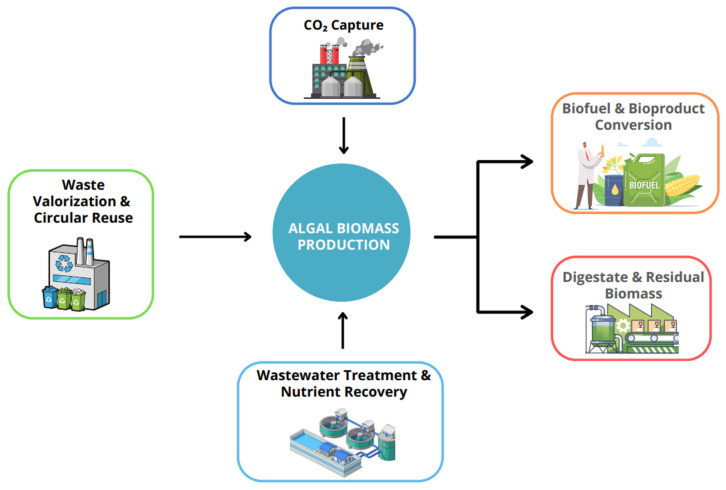
Circular bioeconomy framework illustrating algal biomass production as a central hub linking CO_2_ capture, wastewater treatment, waste valorization, and the generation of biofuels and bioproducts. Arrows indicate the directional flow of resources and conversion pathways, while different colors are used to distinguish key functional processes within the circular bioeconomy system. This figure is an original creation by the authors, developed specifically for this manuscript based on published literature data [129,131]. The figure was created using Canva https://www.canva.com/design/DAG7zh42kNM/1L2Y631MD10_FZZcN6TkzQ/edit?utm_content=DAG7zh42kNM&utm_campaign=designshare&utm_medium=link2&utm_source=sharebutton (accessed on 18 December 2025). Algal-based systems play a dual role in circular bioeconomy models: (i) biological carbon capture and utilization through photosynthesis and (ii) waste valorization via the recovery of nutrients from wastewater and industrial effluents. When integrated into biorefinery concepts, algal biomass can be fractionated into lipids, carbohydrates, and proteins, supporting the simultaneous production of bio-oil, biofuels, and bioproducts, thereby improving overall process efficiency and resilience [129].

The circular bioeconomy (Figure 7) operates on the principles of minimizing waste and optimizing resource reuse through a closed-loop system, linking raw material extraction, sustainable product design, production and remanufacturing, consumption, and waste management [129]. This model contrasts sharply with the traditional linear “take–make–dispose” economy by reintroducing waste into the value chain as a secondary resource, thereby reducing both material losses and environmental impacts. In the European Union, however, only about 12% of materials are currently recycled back into the economy, indicating substantial room for improvement [130].

The U.S. Department of Energy (DOE) has set an ambitious goal to reduce the production cost of algal biofuels to USD 2.50 per gallon by 2030, which would make them competitive with fossil fuels. However, techno-economic analyses indicate that the minimum selling price (MSP) of algal biodiesel still ranges from USD 5.89 per liter to as high as USD 30 per kilogram for hydrogen derived from microalgae, depending on strain, cultivation method, and conversion technology [132].

Similarly, Chen [133] demonstrated that enzymatic hydrolysis followed by fermentation for bioethanol production yielded a minimum ethanol price of USD 548 per ton, with significant potential for cost reduction through integrated process optimization.

Despite these cost challenges, algae remain one of the most promising platforms for decarbonized energy production and waste circularity. When cultivated on nutrient-rich wastewater, algal systems not only sequester CO_2_ but also recover nitrogen and phosphorus, producing biomass for subsequent biofuel conversion [134].

In Japan, the Algae Industry Incubation Consortium has emphasized the use of algal biomass as an alternative feedstock for bio-based chemicals and energy carriers, contributing to fossil fuel displacement and industrial decarbonization.

The shift from a fossil-fuel-based linear economy to a circular bioeconomy built on algal biotechnology has multiple environmental benefits. It reduces GHG emissions, including CO_2_ and N_2_O, that are key drivers of global warming and ozone depletion [133]. Moreover, bioeconomic systems can effectively manage organic and inorganic waste, transforming it into renewable energy and valuable bioproducts while enhancing national energy security [135].

To establish resilient algal-based ecosystems, multidisciplinary integration is essential, combining biotechnology, systems ecology, and policy frameworks to ensure scalability and long-term economic viability. These systems embody the principle of “waste-to-value,” transforming carbon emissions and waste effluents into sustainable commodities. As global bioeconomy initiatives continue to expand, algal biotechnology stands at the core of this transformation, bridging energy production, environmental restoration, and sustainable development.

Unlike linear fossil-based systems, circular algal biorefineries emphasize resource efficiency, waste minimization, and multi-product valorization. This approach aligns with global sustainability initiatives and policy frameworks that prioritize low-carbon technologies and renewable resource utilization [136,137,138]. Although economic and technical challenges remain, particularly with respect to scale-up and process integration, algal biofuels continue to attract attention as key contributors to decarbonized energy systems.

In this framework, algal biotechnology serves as a connecting element between energy production, wastewater management, and carbon mitigation. By transforming waste streams into renewable energy carriers and bioproducts, algal-based circular systems offer a viable pathway toward sustainable development and long-term energy resilience.

## 7. Conclusions

Biofuels derived from phototrophic microorganisms represent a promising pathway toward low-carbon and resource-efficient energy systems. Microalgae and cyanobacteria offer unique advantages as biofuel feedstocks due to their high productivity, capacity for carbon fixation, and compatibility with diverse cultivation environments, including wastewater streams. However, their successful deployment depends on the effective integration of biological optimization, thermochemical conversion, and downstream upgrading strategies.

This review highlights that no single conversion route or technological solution is sufficient to achieve sustainable algal biofuel production. Instead, progress relies on combining hydrothermal liquefaction, pyrolysis, and catalytic upgrading with targeted biotechnological interventions, such as metabolic engineering, co-cultivation, and process intensification. The integration of these approaches within biorefinery frameworks enables improved energy efficiency, enhanced fuel quality, and better utilization of waste resources.

Future research should focus on addressing key bottlenecks related to scale-up, process integration, and economic feasibility. In particular, advances in systems-level optimization, real-time monitoring, and data-driven control strategies, coupled with supportive policy and regulatory frameworks, will be critical for translating laboratory-scale developments into industrial applications. By aligning technological innovation with circular bioeconomy principles, phototrophic microorganisms can play a significant role in the transition toward sustainable and resilient bioenergy systems.

## Figures and Tables

**Table 1:** Comparative overview of bio-oil production methods from phototrophic microorganisms.

Method	Operating Conditions	Feedstock Type	Main Advantages	Limitations	Bio-Oil Yield and Quality	References
Hydrothermal Liquefaction (HTL)	300–400 °C; 180–300 bar; aqueous medium	Wet microalgae and cyanobacteria (70–90% moisture)	Direct use of wet biomass (no drying); high energy density (up to 46 MJ/kg); short reaction time	High pressure requirement; product upgrading needed due to N- and O-containing compounds	45–60% yield; Energy dense, viscous oil with moderate oxygen content	[57,58]
Fast Pyrolysis	450–600 °C; oxygen-free; 100–1000 °C/s heating rate	Dry or lipid-rich algal biomass	Simple process; high oil yield (50–60%); scalable reactors (fluidized-bed)	Requires dry biomass; high O_2_ and H_2_O content; oil instability	50–62% yield (dry basis); oxygen-rich bio-oil requiring upgrading	[59,60]
Catalytic Pyrolysis	450–550 °C with catalysts (HZSM-5, ZrO_2_, Al_2_O_3_)	Lipid-rich algal biomass	Produces aromatics (BTX compounds); improved stability	Reduced overall yield; catalyst deactivation over time	36–40% aromatics; reduced oxygen and nitrogen content	[61,62]
Catalytic Hydroprocessing (HDO)	200–400 °C; ≥30 bar H_2_; catalysts (Ni–Mo, Co–Mo, Pt, Pd, Ru)	HTL or pyrolysis bio-oil	Removes oxygen/nitrogen; increases fuel stability; produces drop-in hydrocarbons	High cost of catalysts and hydrogen; complex reactor design	>80% O removal; high calorific value; hydrocarbon fuel-like oil	[63,64]
Biotechnological Approaches	Ambient; nutrient/stress-induced lipid accumulation; gene overexpression	Engineered or native microalgae	Increases lipid productivity (up to 2×); enhances CO_2_ fixation; environmentally friendly	Long development time; scale-up challenges	Lipid content up to 60–70% dry weight; improved fatty acid composition	[65,66]

**Table 2:** Representative biotechnological and process-intensification strategies enhancing bio-oil production from phototrophic microorganisms.

Process Strategy	System	Underlying Mechanism	Outcome	References
SAMS overexpression	*Chlamydomonas reinhardtii*	Enhanced growth and lipid accumulation	1.5–2× lipid increase	[77]
HSbZIP1 transcription factor	*Chlorella* sp. HS2	Increased fatty acid content	~2× fatty acids	[78]
ACCase/DGAT overexpression	Engineered microalgae	Redirected carbon flux to lipids	+142% lipid accumulation	[79]
Co-cultivation	*C. reinhardtii* + *A. chroococcum*	Improved lipid productivity	19× increase	[80]
Cavitation pretreatment	Microalgae biomass	Enhanced lipid extraction	Reduced solvent and energy demand	[81,82]
Supercritical alcohol blending	Algal bio-oil	Improved stability and HHV	Reduced acidity; higher HHV	[8,83,84]

**Table 3:** Nutrient removal efficiency and biomass productivity of microalgae species cultivated in wastewater streams.

Microalgal Species	Wastewater Type	Nitrogen Removal (%)	Phosphorus Removal (%)	Biomass Productivity (g/L/day)	References
*Chlorella vulgaris*	Municipal wastewater	85–92	78–82	0.35–0.45	[96]
*Scenedesmus obliquus*	Domestic wastewater	80–88	70–76	0.30–0.40	[97]
*Auxenochlorella protothecoides*	Industrial effluents	75–85	65–70	0.28–0.38	[100]
*Ankistrodesmus* *falcatus*	Agro-industrial wastewater	82–90	73–79	0.32–0.44	[101]
*Dunaliella* *tertiolecta*	Synthetic nutrient medium	88–91	77–83	0.36–0.47	[102]

## Data Availability

The original contributions presented in the study are included in the article. Further inquiries can be directed to the corresponding authors.

## Abbreviations

The following abbreviations are used in this manuscript:

CBP	Consolidated Bioprocessing
CCM	Carbon-Concentrating Mechanism
CCGT	Combined Cycle Gas Turbine
CCU	Carbon Capture and Utilization
CO_2_	Carbon Dioxide
CRISPR	Clustered Regularly Interspaced Short Palindromic Repeats
DGAT	Diacylglycerol Acyltransferase
DME	Dimethyl Ether
DOE	U.S. Department of Energy
FAME	Fatty Acid Methyl Esters
FDA	Food and Drug Administration
GDP	Gross Domestic Product
GHG	Greenhouse Gas
HHV	Higher Heating Value
HTL	Hydrothermal Liquefaction
IPCC	Intergovernmental Panel on Climate Change
ME	Metabolic Engineering
ME-Model	Integrated Model of Metabolism and Macromolecular Expression
MSP	Minimum Selling Price
PBR	Photobioreactor
RSM	Response Surface Methodology
SAR	Structure–Activity Relationship
SCF	Supercritical Fluid
SDGs	Sustainable Development Goals
SHF	Separate Hydrolysis and Fermentation
SO_x_	Sulfur Oxides
TAG	Triacylglycerol
TEA	Techno-Economic Analysis
TO	Thermodynamic Optimization
VFA	Volatile Fatty Acids
